# Reduced expression of the proteolytically inactive FtsH members has impacts on the Darwinian fitness of *Arabidopsis thaliana*

**DOI:** 10.1093/jxb/erz004

**Published:** 2019-02-04

**Authors:** Laxmi S Mishra, Kati Mielke, Raik Wagner, Christiane Funk

**Affiliations:** Department of Chemistry, Umeå University, Umeå, Sweden

**Keywords:** AAA-type protease, embryo lethal, field trial, FtsH metalloprotease, phenotype, seedling

## Abstract

FtsH (filamentation-temperature-sensitive protein H) proteases are a family of membrane-bound enzymes present in eubacteria, animals, and plants. Besides the 12 genes encoding proteolytically active members of the FtsH family in the genome of Arabidopsis, there are five genes coding for members that are assumed to be proteolytically inactive due to mutations in the protease domain; these are termed FtsHi (i for inactive). Despite their lack of proteolytic activity, these FtsHi members seem to be important for chloroplast and plant development as four out of five homozygous knockout-mutants of FtsHis are embryo-lethal. Here, we analysed the Darwinian fitness of weak homozygous (*ftshi1,3,4*) and heterozygous (*ftshi/FTSHi2,4,5*) mutants. We compared the growth and development of these mutants to their respective wild-type Arabidopsis plants under controlled laboratory conditions and in the field, and we also evaluated the photosynthetic efficiency by pulse-amplitude modulation fluorescence. Homologous genotypes were subjected to various stress conditions in a greenhouse and gene co-expression as well as phylogenetic analyses were performed. Analysis of the gene-expression network of the five *FTSHi* genes indicated common clusters with genes encoding FtsH12, OTP51, and methylase. Phylogenetic analyses pointed to a common evolution (and common disappearance in grasses and gymnosperms) of FtsH12 and multiple presumably proteolytically inactive FtsHi enzymes. Our data show that the FtsHi enzymes are highly important during the seedling stage and for Darwinian fitness analyses in semi-natural conditions.

## Teaser

The evolution of multiple genes encoding presumably proteolytically inactive filamentation-temperature-sensitive protein H (FtsHi) proteases accompanied the evolution of *FTSH12*, and Arabidopsis mutants with reduced *FTSHi* expression show decreases in Darwinian fitness.

## Introduction

FtsH (filamentation-temperature-sensitive protein H) proteases are a family of membrane-bound metalloproteases present in eubacteria, animals, and plants. These proteases consist of an AAA (ATPase associated with various cellular activities) domain and a metalloprotease domain ligating a Zn^2+^ ion in the consensus sequence HEXXH (where X is any uncharged residue). They are inserted into the membrane with one or more transmembrane helices located at the N-terminus of the enzyme (reviewed in Wagner *et al.*, 2012; Kato and Sakamoto, 2018). The conserved AAA domain is characterized by an ATP-binding motif (Walker A and Walker B) and the second region of homology (SRH) needed for ATP cleavage (Karata *et al.*, 1999). AAA/FTSH proteins are known to form functional homo- or hetero-hexameric complexes (Bieniossek *et al.*, 2006). As a result of ATP-driven hydrolysis in the AAA-domain, these complexes are able to switch between an open and closed status by moving a conserved hydrophobic area (FGV pore motif) towards the interior proteolytic chamber (Bieniossek *et al.*, 2009); an opened state is required for protease activity.

While the genome of the bacterium *Escherichia coli* only contains one *ftsh* gene, multiple copies of this protease can be found in organisms performing oxygenic photosynthesis; for example, the cyanobacterium *Synechocystis* sp. PCC 6803 contains four FtsH proteins and 17 different FtsH proteases have been identified in Arabidopsis (Wagner *et al.*, 2012). The subcellular localization of eukaryotic FtsHs is restricted to organelles of endosymbiotic origin (mitochondria and chloroplasts). In Arabidopsis, 13 FtsH enzymes are localized in the chloroplast, whilst three are targeted to mitochondria. FtsH11 was reported to be dual-targeted to both organelles (Urantowka *et al.*, 2005); however, recent biochemical studies on 6-week-old plants confirmed its location only in chloroplasts (Wagner *et al.*, 2016). Five of the plastid-located FtsHs have been identified in the thylakoid membrane; of these, FtsH1, 2, 5, and 8, are highly expressed and form well-studied heteromeric complexes important for the degradation and assembly of D1 proteins and other transmembrane subunits of the photosynthetic machinery (reviewed in Putarjunan *et al.*, 2013; Kato and Sakamoto, 2018). FtsH6, also located in the thylakoid membrane, has recently been shown to be involved in thermomemory of seedlings of Arabidopsis (Sedaghatmehr *et al.*, 2016), although adult plants do not show any phenotype when grown in semi-natural outdoor conditions (Wagner *et al.*, 2011). The remaining plastid-located FtsH enzymes (FtsH7, 9, 11, 12, FtsHi1–5) have been detected in the chloroplast envelope using proteomics approaches (Ferro *et al.*, 2010).

Interestingly, in five of these plastidic FtsH enzymes of Arabidopsis either the histidine residues of the conserved protease domain are mutated (FtsHi1, 2, 4, and 5) or the whole HEXXH motif is missing (FtsHi3), rendering them (presumably) proteolytically inactive (Wagner *et al.*, 2011). Hence these enzymes are termed FtsHi (i for inactive). Their location in the plastid envelope has been confirmed for FtsHi1 (also called ARC1) (Kadirjan-Kalbach *et al.*, 2012); FtsHi4, however, has been shown to be located in the thylakoid membrane (Lu *et al.*, 2014). FtsHi1/ARC1 seems to be important for chloroplast division, biogenesis, and embryogenesis in Arabidopsis (Kadirjan-Kalbach *et al.*, 2012). While a *ftshi1-2* mutant is embryo-lethal, the ‘weak’ *ftshi1-1* mutant displays a pale seedling phenotype compared to the wild-type (Kadirjan-Kalbach *et al.*, 2012). This mutant contains an increased number of small chloroplasts as a result of accelerated or premature plastid division, and young seedlings of *ftshi1*/*arc1* contain wavy, swollen, and less-organized thylakoid membranes with reduced de-etiolation efficiency (Kadirjan-Kalbach *et al.*, 2012). Mutants depleted of FtsHi4 display embryo-lethality and disrupted thylakoid formation in Arabidopsis, leading to reduced PSII function during embryogenesis (Lu *et al.*, 2014). Impairment of FtsHi5 (PR1, photorespiratory-related 1) has been shown to affect photorespiration in Arabidopsis and to increase senescence; the enzyme might be important for the cellular redox balance (Wang *et al.*, 2018). Four out of the five presumably proteolytically inactive FtsH enzymes have been shown to form a hetero-hexameric AAA-ATPase complex with the FtsH12 protease and to co-localize with plastidic malate dehydrogenase (pdMDH) and the hypothetical chloroplast open reading frame 2 (Ycf2) (Kikuchi *et al.*, 2018; Schreier *et al.*, 2018). This protease complex seems to play an important role in chloroplast development. Knock-out of *FTSH12* in Arabidopsis results in embryo lethality (Schreier *et al.*, 2018); similar knock-outs of *FTSHi 2*, *4*, and *5* are embryo-defective.

To investigate the impact of the presumably proteolytically inactive FtsHi enzymes in Arabidopsis and to determine their functions, we performed *in silico* and *in vivo* analyses. We evaluated phenotypes as well as photosynthetic parameters of homozygous and heterozygous *FTSHi* mutants exposed to various stress conditions and grown in the field (outdoor experiment). Being not seed-lethal, these weak homozygote or heterozygote mutants provide a good opportunity to learn more about the role of FtsHi enzymes in higher plants. Analysis of the gene-expression network of the five *FTSHi* genes indicated common clusters with genes encoding FtsH12, OTP51, and methylase family protein (mraW). Phylogenetic analyses pointed to a common evolution (and common disappearance in grasses and gymnosperms) of FtsH12 and multiple presumably proteolytically inactive FtsHi enzymes. Darwinian fitness analyses in semi-natural conditions indicated that the FtsHi enzymes are highly important during the seedling stage.

## Material and methods

### Plant material and growth conditions

*Arabidopsis thaliana* wild-types (Col-0, Col-3, L*er*) and EMS and T-DNA mutant seeds were obtained from The Nottingham Arabidopsis Stock Centre (NASC, http://arabidopsis.info/); their genotypes were verified by sequencing-based methods and are listed in Supplementary Table S1 at *JXB* online. Primers used for genotyping are listed in Supplementary Table S2.

After sterilization with 10% sodium hypochlorite followed by four washes with sterile water and stratification for 48 h at 4 °C, the seeds were selected on full-strength Murashige and Skoog (MS) agar plates (Murashige and Skoog, 1962), supplemented with 1% sucrose and respective antibiotics. After growing for 10 d on plates the plants were transferred to soil.

Outdoor experiments in Umeå, Sweden (63°49´07.2´´N 20°18´45.0´´E) were performed during the weeks 26–36 in 2017 as described by Frenkel *et al.* (2008) and Wagner *et al.* (2012). Plants were grown under long-day (LD) conditions (16/8 h light/dark, at 21 °C) in a greenhouse for 10–12 d, acclimatized for 24 h outdoors, and then transferred to natural outdoor conditions. The experiment was performed with two lines and 50 plants per *FTSHi* genotype. The mutants and their respective wild-type controls were randomly arranged and placed in the field, where the plants were exposed to fluctuating temperature, water supply, and light up to high intensities (1500–2000 µmol photons m^−2^ s^−1^). At this latitude the photoperiod equals continuous light during the summer months; temperature, humidity, rainfall and solar radiation were measured at Umeå University (http://www.tfe.umu.se). Weather data collected at a station 650 m from the growth site can be obtained from the Department of Applied Physics and Technology, Umeå University, Sweden (http://www8.tfe.umu.se/weather-new/hamta_vaderdata.html). The vegetative growth of the plants was monitored by measuring their rosette diameter (10 replicates per line) at intervals of 3–4 d, until the plants reached an age of 40 d. After 10 weeks in the field, the plants were transferred indoors for drying seeds and siliques to be counted.

For the stress experiments, plants were grown in a growth chamber under the following conditions: (1) short days (SD), 8/16 h light/dark, at 22 °C; (2) SD with low temperature, at 4 °C; (3) SD with high light, at 700 µmol photons m^−2^ s^−1^ at 22 °C; (4) long days (LD), 16/8 h light/dark, at 22°C; (5) LD with high temperature, at 30 °C; and (6) continuous light. Unless stated otherwise, the light intensity was maintained at 150 µmol photons m^−2^ s^−1^, and the relative humidity was 70%. The rosette size of 10 randomly chosen replicates per line was measured at intervals of 3–4 d until the plants reached an age of 40 d.

### Phenotypic characterization

Seedlings of the wild-type and FtsHi mutants were examined using a Leica MZ9.5 Stereomicroscope at days 4, 6, and 8 after germination. Plants grown in the field or exposed to stress conditions were photographed when they were 6 weeks old using a Canon 650D camera.

### Determination of chlorophyll content

Chlorophyll *a* and *b* from whole 2-week-old seedlings (cotyledons and true leaves) were extracted and calculated per mg leaf fresh weight according to Porra *et al.* (1989).

### Chlorophyll fluorescence parameters

Chlorophyll fluorescence was measured using the pulse-amplitude-modulation technique (PAM) with a PAM-210 fluorometer (Walz, Germany) according to the manufacturer’s instructions. 

For outdoor and stress experiments, 10 independent biological replicates of 6-week-old whole plants were dark-adapted for 30 min and the maximum PSII quantum yield, *F*_v_/*F*_m_, was recorded in the 8th leaf. The total measuring time was 120 s, with saturation pulses of 800 ms; data were collected every 10 s. A 1-s light saturation pulse of ~3000 mol m^–1^ s^–1^ was used to record *F*_0_.

### RNA extraction and quantitative real-time PCR (qRT-PCR)

Total RNA was extracted from young leaves of three biological replicates of 6-week-old wild-types, EMS and T-DNA mutants using a RNAqueous Total RNA Isolation Kit (Invitrogen). Quantitative RT-PCR was performed using a BioRad CFX96 thermocycler. The primers used for the housekeeping genes (*ubiquitin* and *actin*) and the gene-specific Q-PCR are listed in Supplementary Table S2. Isolated RNA was reverse-transcribed into cDNA using a RevertAid First Strand cDNA Synthesis Kit (Thermo Scientific). The data were analysed using the BioRad CFX Manager 3.1 software.

### Co-expression studies

*In silico* gene co-expression data from the publicly available database Atted-II version 9.2 (http://atted.jp/) was analysed, and visualized using the Graphviz software (https://www.graphviz.org/). A set of six query genes (five assumed to be proteolytically inactive FtsHis and the proteolytically active FtsH12) were subjected to the Atted-II Network Drawer output co-expression network for Arabidopsis (Ath-m). The threshold (minimum of three genes strongly co-expressed within the query set) was selected to ‘Add few genes’ and ‘Draw PPI’ (protein–protein interactions). The genes directly linked to at least one of the proteolytically inactive FtsH proteases were kept in the network.

### Fitness and statistical analyses

Darwinian fitness in the outdoor experiments was evaluated by determining the number of surviving plants of a particular genotype. In addition, the mean number of seeds produced in five siliques per plant and the total number of siliques were determined. Harvested seeds from three independent plants per genotype were plated on full-strength MS medium agar plates to assess their viability and germination capacity. Statistical analyses were performed using the SPSS software. All *P*-values were determined using ANOVA and a least-significant difference (LSD) *post hoc* test.

### Phylogenetic analyses

The FtsH12 protein sequence was retrieved from UNIPROT (ID Q9SAJ3, www.uniprot.org) and searched in Phytozome (http://phytozome.jgi.doe.gov, version 12.1.6) with blastp (Altschul *et al.*, 1990), with expected e-threshold –1, comparison matrix BLOSUM62, word length 3, and gaps allowed. The following species were investigated in Phytozome: *Chlamdomonas reinhardtii*, *Dunaniella salina*, *Physcomitrella patens*, *Selaginella moellendorfii*, *Spirodela polyrhiza*, *Oryza sativa*, *Setaria italica*, *Zea mays* PH207, *Aquilegia coerula*, *Solanum lycopersicum*, *Glycine max*, *Populus trichocarpa*, and *Arabidopsis thaliana*. Highest bit scores from blastp and the identity % were noted. All proteins similar to FtsH12 were manually investigated for the zinc binding motif HEXXH and the presence of an AAA domain via Phytozome or InterProScan (https://www.ebi.ac.uk/interpro/search/sequence-search). Proteins lacking the HEXXH motif were assumed to be proteolytically inactive (FtsHi). The Phytozome database does not cover gymnosperms or cyanobacteria, and therefore sequence comparisons for cyanobacterial species (*Prochlorococcus marinus*, *Synechocystis* sp. *PCC803*, and *Anabaena cylindrica*) were performed on NCBI (https://blast.ncbi.nlm.nih.gov) with blastp and standard parameters: expected threshold 10, word size 6, comparison matrix BLOSUM62, gap cost 11, gap extension 1, and conditional compositional score matrix adjustment. FtsH proteins were further searched in the gymnosperm *Picea abies* by comparing the FtsH12 sequence in its proteome database on Congenie (http://congenie.org) by blastp. Genes of high and medium confidence were selected. Standard blastp parameters on Congenie were used: comparison matrix BLOSUM62, e-value cut-off 1e^−3^, gapped, no frame shift penalty. Data for *C. reinhardtii* and *O. sativa* were supplemented with data from the literature (Garcia-Lorenzo *et al.*, 2008; Malnoë *et al.*, 2014). Ycf2, which is plastid-encoded, was searched for in NCBI organelle genome resources (https://www.ncbi.nlm.nih.gov/genome/organelle/).

## Results

### The presence of multiple FtsHis evolved together with FtsH12

**Fig. 1.**
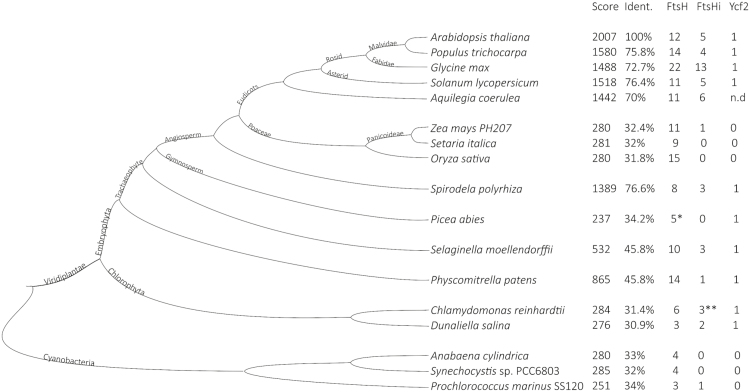
Co-evolution of FtsH12 and FtsHi. The FtsH12 amino acid sequence was blasted via Phytozome. Hits with the highest score and identity are shown. The number of presumably proteolytically active (FtsH) and -inactive (FtsHi) genes were determined by manually investigating the presence of an AAA-like domain and peptidase M41 domain. Sequences containing a zinc-binding motif (HEXXH) within M41 were assumed to correspond to proteolytically active proteases. Data for *Picea abies* were additionally retrieved from Congenie (marked with asterisks). Data for *Prochlorococcus*, *Synechocystis*, and *Anabaena* were retrieved from NCBI. Data for *Chlamydomonas reinhardtii* and *Oryza sativa* were supplemented with data from the literature (Garcia-Lorenzo *et al*., 2008, Malnoë *et al.*, 2014). Ycf2 is plastid-encoded and therefore the search was performed on plastid-containing organisms, except for *Aquilegia*, in the NCBI organelle genome resources.

FtsHi1 (AT4G23940), FtsHi2 (AT3G16290), FtsHi3 (AT3G02450), FtsHi4 (AT5G64580), and FtsHi5 (AT3G04340) are annotated by TAIR (http://arabidopsis.org) as members of the FtsH family containing the AAA-ATPase domain (IPR003593), the ATPase-AAA core (IPR003959), and the M41 peptidase domain (IPR000642). Interestingly, the consensus sequence HEXXH of the M41 peptidase metalloprotease domain present in active FtsH proteases is either mutated (FtsHi1, 2, 4, and 5) (Zhang *et al.*, 2010) or entirely missing (FtsHi3) in the FtsHis of Arabidopsis (Supplementary Fig. S1), rendering them presumably proteolytically inactive. In addition, in FtsHi3 the M41 peptidase domain is located before the N-terminal instead of the C-terminal of the AAA-ATPase. All FtsHis are predicted to contain a chloroplast transit peptide at the N-terminus (http://www.cbs.dtu.dk/services/TargetP/) (Emanuelsson *et al.*, 2007) followed by three (FtsHi1, 5), two (FtsHi2, 3), or one (FtsHi4) transmembrane domains (http://www.cbs.dtu.dk/services/TMHMM/) (Supplementary Fig. S1). FtsH peptidases are core elements of the proteolytic machinery in eubacteria and eukaryotic organelles. While there is only one gene copy in the enterobacterium *E. coli*, the genomes of simple cyanobacteria already contain four copies (Shao *et al.*, 2018, Fig. 1). Arabidopsis contains 17 FtsH proteases, of which 12 have a zinc-binding HEXXH motif in their peptidase M41 domain. Based on the recent finding that some FtsHis in Arabidopsis form a complex together with AtFtsH12 (Kikuchi *et al.*, 2018; Schreier *et al.*, 2018), the protein sequence of FtsH12 (Uniprot ID: Q9SAJ3) was blasted against the translated genomes of various species of cyanobacteria, green algae, and higher plants using the Phytozome resource, NCBI (for cyanobacteria), and Congenie (for *Picea abies*). The highest BLAST scores and percent of amino acid identities of the covered sequence compared to AtFtsH12 as well as the number of FtsH and FtsHi proteins identified in each species are shown in Fig. 1. While the chlorophyll *a*-binding cyanobacteria (as well as non-photosynthetic organisms; data not shown) did not contain any FtsHi, interestingly one FtsHi was observed in *Prochlorococcus marinus* SS120, a chlorophyll *b*-containing cyanobacterium with a highly reduced genome. In green algae, the copy number of both FtsH and FtsHi was found to be low (Fig. 1), whilst in other algal phyla (e.g. cryptophytes, diatoms) FtsHi enzymes seemed to be absent (data not shown). Similar results were obtained when performing a search with full-length AtFtsHi1 instead of AtFtsH12 (Supplementary Table S3). *FTSH* genes in these evolutionary ‘old’ organisms showed low similarities to FtsH12, with scores ranging from 250–285 and identities between 30–35%. With the evolution of land plants, i.e. in the moss *Physcomitrella patens* and the fern *Selaginella*, copy numbers of FtsH as well as scores towards AtFtsH12 increased, while the number of FtsHi members was found to remain low. Interestingly, the number of FtsHis also seemed to be low in gymnosperms (e.g. *Picea abies*), while duckweed (*Spirodela polyrhiza*) contained FtsHi (three FtsHis were found using AtFtsH12 and six using AtFtsHi1 in the search). In this flowering plant, not only did the total number of FtsH proteases increase, but the presence of FtsH12 also seemed to occur for the first time in evolution (*S. polyrhiza* contained an FtsH protease with 76.6% identity to FtsH12). While FtsH12 was missing and the number of FtsHis was low in monocotyledons (Fig. 1), in eudicots the number of FtsHi enzymes together with the scores and identities towards AtFtsH12 strongly increased.

According to Kikuchi *et al.* (2018), the chloroplast-encoded Ycf2 is closely related to FtsHis. We therefore performed an additional search for the presence/absence of Ycf2 in our selected species using the NCBI organelle genome resources for all eukaryotes. Based on these data, Ycf2 seemed to be absent in monocotyledons, but was present in all other eukaryotic organisms (Fig. 1).

### Co-expression analysis and expression in mutants identifies co-regulation of FTSHi

**Fig. 2.**
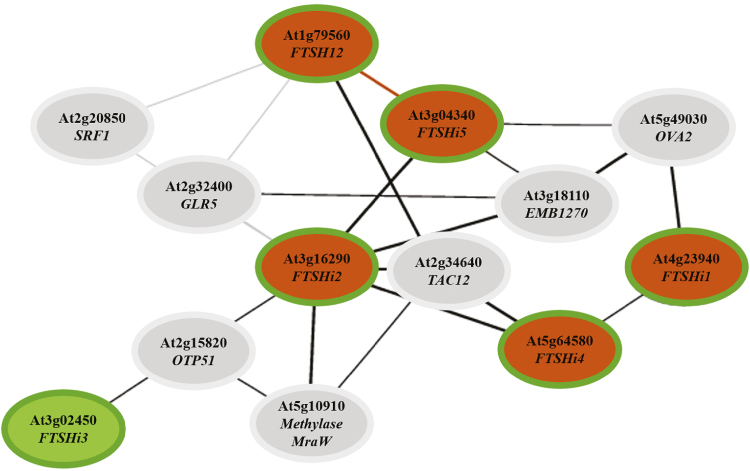
Co-expression network of the five *FTSHi* genes (green borders) in Arabidopsis. Co-expressed genes are connected by lines as follows: bold lines, mutual rank (MR) <5; black lines, MR<30; thin grey lines, MR≥30; orange lines, predicted protein–protein interaction. Orange shading indicates an identified protein–protein interaction (Kikuchi *et al.*, 2018; Schreier *et al.*, 2018). The co-expressed genes and their corresponding protein names and localizations predicted by TargetP and WoLF PSORT as identified in ATTED-II are listed in Supplementary Table S4.

Genes that are co-expressed during certain environmental or developmental stimuli might encode proteins that are involved in the same metabolic pathways or that interact with each other. To obtain clues about the biological function of the five FtsHi enzymes, publicly available gene expression databases were examined to infer a co-expression network (Fig. 2). The NetworkDrawer in ATTED-II v. 9.2 has a threshold of drawing edges for a minimum of three genes with the strongest co-expression within the query set (Obayashi *et al.*, 2018). Co-expressed genes identified in ATTED-II and their corresponding proteins and localizations predicted by TargetP and WoLF PSORT are listed in Supplementary Table S4. Based on the results obtained, *FTSHi*2 and *FTSHi*5 were strongly co-expressed, as were *FTSHi2* and *FTSHi4*, whilst *FTSHi4* and *FTSHi1* were also co-expressed but to a weaker extent (Fig. 2). In addition, the co-expression of *FTSHi*5 and *FTSH12* was strong, and their corresponding proteins were even predicted to interact with each other. *FTSHi*2, *FTSHi4*, and *FTSH12* also strongly correlated with a gene encoding TAC12 (AT2G34640), a component of the plastidic transcription complex, which along with other TAC proteins has been shown to play an important role in phytochrome-dependent signalling during plastid development (Chen *et al.*, 2010; Lu *et al.*, 2014). *FTSHi1* and *FTSHi*5 were linked by a gene encoding OVA2 (AT5G49030), known as ovule abortion. Immature siliques of *ova* mutants display small, white structures that resemble unfertilized ovules amongst normal seeds at the cotyledon stage of embryo development (Berg *et al.*, 2005). The expression of *FTSHi*3 did not correlate with the tight cluster of *FTSHi1*, *2*, *4*, *5* and *FTSH12*, and instead it co-expressed with a gene encoding OTP51 (AT2G15820), a pentatricopeptide repeat protein that is required for the splicing of group-IIa introns and which impacts on PSI and PSII assembly (De Longevialle *et al.*, 2008). *FTSHi2* was also co-expressed with this gene.

To investigate the function of the presumably proteolytically inactive FtsHi enzymes of Arabidopsis, mutants were screened for homozygous knock-out lines from a collection of heterozygous T-DNA insertion lines. The EMS mutation for *ftshi1-1* in the *ARC1*/*FTSHi1* gene is a missense mutation that alters the first amino acid of the Walker B motif (S524F) (Kadirjan-Kalbach *et al.*, 2012). *Ftshi1-1* was selected and the mutation was confirmed by sequencing. The other *FTSHi* mutants used were T-DNA insertion mutants (Supplementary Fig. S2, Supplementary Table S1) that were self-pollinated, and their progenies were genotyped. As well as *ftshi1-1*, five T-DNA insertions lines in *FTSHi2*, one line in *FTSHi3*, two lines in *FTSHi4*, and two lines with insertions in *FTSHi5* were screened for their genotypes and phenotypes. The T-DNA insertions in *FTSHi2* (*ftshi2-1*, SALK_054712C; *ftshi2-2*, WISCDSLOX383F4; *ftshi2-3*, emb2083-3; *ftshi2-3*, emb2083-4; *ftshi2-4,* emb2083-2; and (*tshi2-5,* SAIL_1178_A11) were located in exons. The *ftshi3-2* (GK_723C06) insertion was located at the intersection of an exon and intron. In *ftshi4-1* (*GK-382B06*) the insertion was located in the intron; however, in *ftshi4-2* (SALK_067969.20.10.x) it was located in the 5´-UTR (Supplementary Fig. S2, Supplementary Table S1). The T-DNA insertions of *ftshi5-1* (GK-058A04) and *ftshi5-2* (emb2458) were located in exons. Whilst *ftshi*1-1, *ftshi3-2*, and *ftshi4-2* were homozygous, the others were found to be heterozygous (Supplementary Table S5). Homozygous mutants of *ftshi4* and *ftshi5* had been previously found to be seed-lethal (Meinke *et al.*, 2008); however, *ftshi4-2* was a weak homozygous mutant, in which gene expression was reduced by 61% (Supplementary Table S5). T-DNA insertions into the promoter or 5´-UTRs have been found to be less effective than insertions in the intron or exon; such a ‘position effect’ is known to result in phenotypic differences (Wang, 2008). In the homozygous mutants *ftshi1-1* and *ftshi3-2*, gene expression was reduced by 88% and 90%, respectively, normalized to the expression in their corresponding wild-type (WT; Supplementary Table S5), whereas the expression in the heterozygous mutants *ftshi*2/*FTSHi2-5*, *2-6*, *4-1*, *5-1*, and 5-2, was reduced by ~40–50% compared to that of the corresponding gene in the WT.

**Fig. 3.**
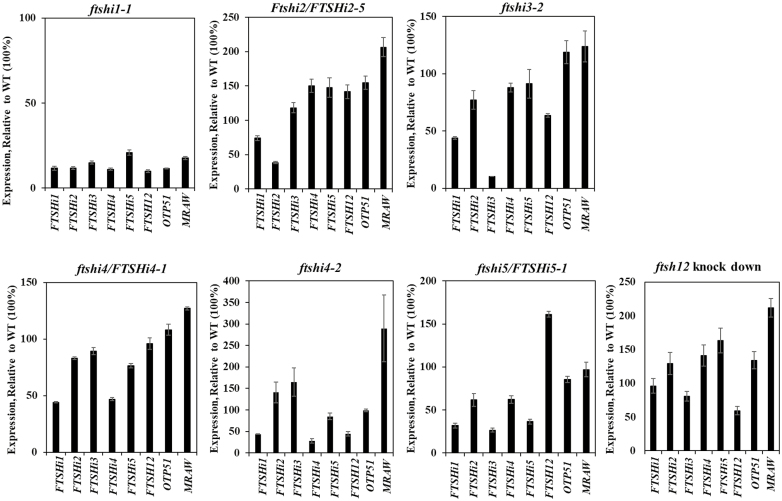
Quantitative real-time PCR to investigate the transcription of Arabidopsis genes encoding *FTSHi1–5*, *FTSH12*, *OTP51*, and methylase family protein *mraW* using mRNA isolated from wild-type and T-DNA mutant plants of *FTSHi1–5 ftsh12* knock-down lines. Three biological replicates were harvested from plants grown under short days. The data were normalized to the expression of genes coding for *UBQ5* and *beta-actin*, and the expression of each gene is shown relative to its expression in the wild-type (100%).

To identify the impact of a particular *FTSHi* mutation on the expression of the other *FTSHi*s, qRT-PCR was performed on RNA extracted from 6-week-old *FTSHi* mutant plants (*ftshi1-1*, *ftshi2*/*FTSHi2-5*, *ftshi3-2*, *ftshi4/FTSHi4-1*, *ftshi4-2*, *ftshi5/FTSHi5-1*), a *ftsh12* knock-down line, and their corresponding WT plants grown under SD conditions (Fig. 3). Expression was investigated for the five *FTSHi* genes, *FTSH12*, *OTP51*, and *mraW*, which were identified in our co-expression studies (Fig. 2). The expression of each gene in relation to its expression in the corresponding WT (100%) is shown in Fig. 3. In the homozygous *ftshi1-1* mutant the expression of the other four *FTSHi*s, *FTSH12*, *OTP51* and the methylase gene was significantly lower than in the WT. The homozygous mutants *ftshi3-2* and *ftshi4-2* displayed significantly lower expression of *FTSHi1* and *FTSH12* compared to the WT, whilst the expression of *mraW* was significantly up-regulated in *ftshi2*/*FTSHi2-5* and *ftshi4-2*. *FTSH12* was highly expressed in *ftshi5/FTSHi5-1*, and conversely expression of *FTSHi5* was found to be significantly increased in the *ftsh12* knock-down line, confirming our ATTED prediction analysis indicating an interaction between the two corresponding proteins.

### Phenotypic characterization of the *FTSHi* mutants indicates functions in development

**Fig. 4.**
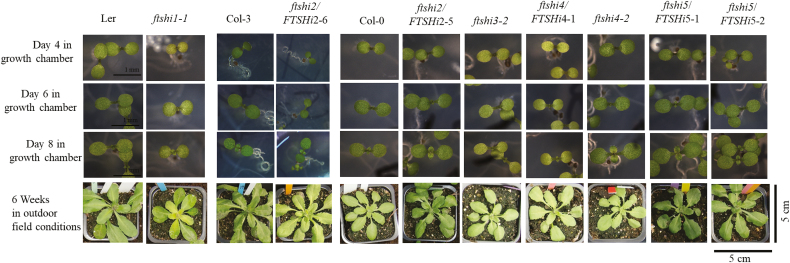
Phenotypes of seedlings of Arabidopsis wild-types (L*er*, Col-3, and Col-0) and the T-DNA mutants of *FTSHi1–5* at 4-, 6-, and 8 d old for plants grown in a growth chamber under short days at 22 °C (scale bars are 1mm), and for 6-week-old plants grown under outdoor field conditions (images are 5×5 cm).

Phenotypic characterization was carried out on the homozygous and heterozygous *FTSHi* lines. The pale-seedling phenotype observed for *ftshi1-1* (Kadirjan-Kalbach *et al.*, 2012) was confirmed, and *ftshi3-2* and *ftshi4/FTSHi4-1* also displayed pale seedlings when grown for 8 d on agar plates (Supplementary Table S5, Fig. 4). Although the pale-seedling phenotype was visible only in younger plants, it was consistent in every generation. Chlorophyll contents and chlorophyll *a*/*b* ratios of the seedlings are given in Supplementary Table S7.

To further investigate the impact of FtsHis, 2-week-old homozygous *FTSHi* mutants (*ftshi1-1*, *ftshi3-2*, *ftshi4-2*) and their respective WTs were subjected to various stress conditions. After 4 weeks of cold stress under short-day (SD) conditions (i.e. plants 6 weeks old), pale-seedling phenotypes differing to the WT were observed for *ftshi1-1*, *ftshi3-2*, and *ftshi4-2* (Supplementary Fig. S3). Interestingly, after exposure for 16 weeks only *ftshi1-1* displayed a phenotype of pale-green leaves and a small rosette(Supplementary Fig. S3A, E). *ftshi1-1* continued to show the small-rosette phenotype at high temperature (30 °C) under long-day (LD) conditions, under continuous light, and under high-light stress in SD . Analyses of maximum PSII quantum yield (*F*_v_/*F*_m_) and non-photochemical quenching (NPQ) were performed on these plants directly after transferring them to stress (*t*=0), and after 3 d, 6 weeks, and 16 weeks (Supplementary Table S6). Significant differences in NPQ were already found between *ftshi1-1* and L*er* before exposure to stress (*t*=0). The *F*_v_/*F*_m_ values of the mutants differed to the WTs only after 3 d of stress exposure, indicating slower stress acclimation; otherwise they were similar to their respective WT controls. Interestingly, in *ftshi3-2* the NPQ values dropped significantly compared to Col-0 after 3 d of stress exposure, in SD/4 °C, LD/30 °C, and continuous light but recovered and were higher than in the WT control after stress exposure for 6 weeks.

### Darwinian fitness of the *FTSHi* mutants

Controlled laboratory growth conditions do not fully mimic the natural environment of plants. Therefore, to investigate the impact of the various FtsHi enzymes, the fitness of the *FTSHi* mutant plants was challenged under natural conditions in Umeå, Northern Sweden in 2017 (Frenkel *et al.*, 2008; Wagner *et al.*, 2012). A summary of the light and temperature conditions at Umeå University located 650 m from the growth site is given in Supplementary Fig. S4.

**Fig. 5.**
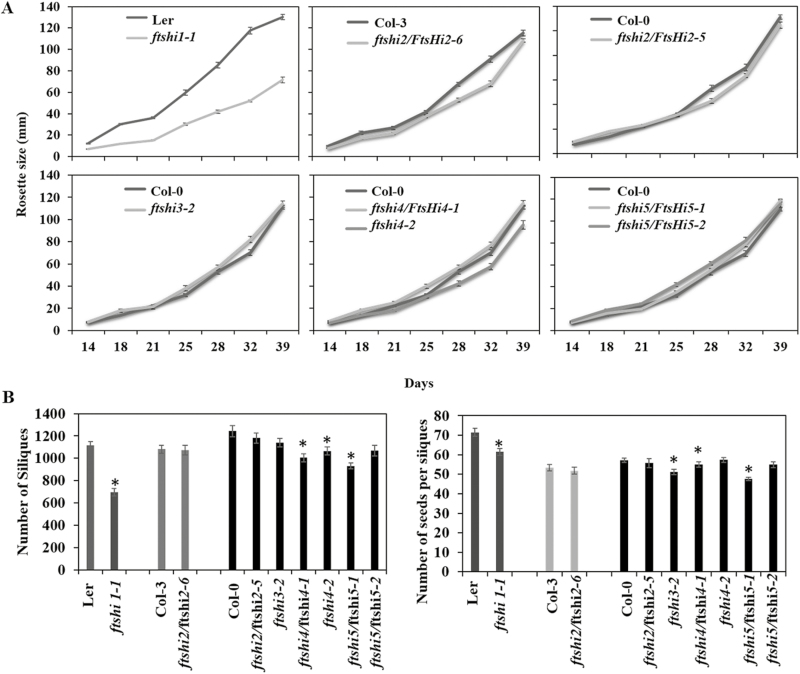
Darwinian fitness analysis of outdoor-grown Arabidopsis *FTSHi* mutant plants and their corresponding wild-types. (A) Rosette diameter measurements were performed from week 2–6. (B) Number of siliques (left) and number of seeds per silique (right). ANOVA and a least significant difference (LSD) *post hoc* test was performed to determine the statistical significance relative to the respective wild-type value (*P*<0.05).

Compared to its wild-type, *ftshi1-1* displayed pale young leaves (Fig. 4, bottom row) after 6 weeks growth in the field, and its rosette size was smaller (Fig. 5A). The leaves of *ftshi3-2*, *4-1*, and *4-2* were also found to be paler, but these mutants were not reduced in growth. Chlorophyll fluorescence parameters were determined in 45-d-old plants and *F*_v_/*F*_m_ did not differ in the mutant lines compared to the WTs (Supplementary Table S8). NPQ was significantly lower in *ftshi1-1* compared to its WT, but it did not differ in the other mutants.

**Fig. 6.**
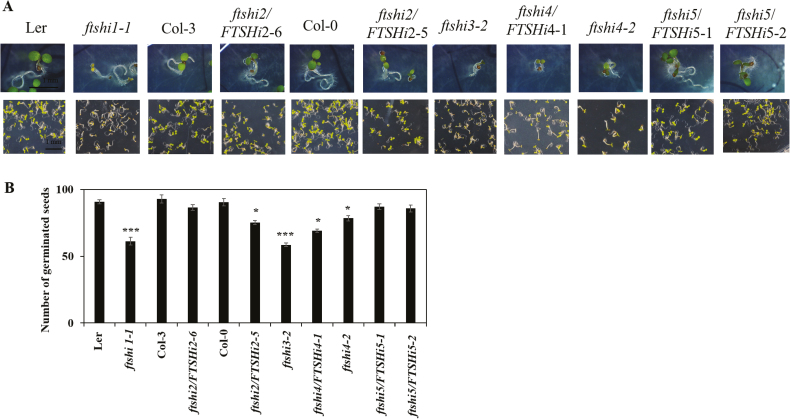
(A) Phenotypes of seeds harvested from field-grown Arabidopsis wild-types (L*er*, Col-3, and Col-0) and T-DNA mutant plants of *FTSHi1–5* at 4 d after germination. Scale bars are 1 mm. (B) Seed germination capacity (determined after 2 d of incubation) of seeds harvested from field-grown plants. ANOVA and a least significant difference (LSD) *post hoc* test and were performed to determine the statistical significance relative to the respective wild-type value: ***P<0.001; *P<0.05.

The experiment was terminated after the first plants presented siliques that were almost dry and ready to shed their seeds. To estimate the Darwinian fitness, the total number of seeds was estimated by counting seeds from five siliques each from 50 plants (Frenkel *et al.*, 2008; Wagner *et al.*, 2011). The total number of siliques was also counted. Due to the high number of replicates per genotype and WT control, the variation amongst the plants was controlled and significant differences were observed in the total number of siliques between L*er* and *ftshi1-1*, and between Col-0 and the *ftshi4*/*FTSHi4-1*, *ftshi4-2*, and *ftshi5/FTSHi5-1* mutants (Fig. 5B). The *ftshi1-1*, *ftshi3-2*, *ftshi4*/*FTSHi4-1*, and *ftshi5/FTSHi5-1* mutants had significantly lower numbers of seeds per silique. The seeds harvested from the plants were subjected to germination tests (Fig. 6A). No significant differences in seed viability were observed between the various *FTSHi* mutants and their respective WTs, despite the pale-green seed phenotypes (Fig. 4); However, a significant delay in seed germination was observed in *ftshi1-1*, *ftshi2*/*FTSHi*2-*6*, *ftshi3-2*, *ftshi4*/*FTSHi4-1*, and *ftshi4-2* compared to their respective WTs after 2 d (Fig. 6B). The germination of *ftshi*1-1 and *ftshi3-2* was strongly affected, indicating lower Darwinian fitness of these mutants.

## Discussion

In this study we investigated five homologues to FtsH proteases of Arabidopsis that, due to mutations in their active site, are presumably proteolytically inactive and therefore termed FtsHi (Wagner *et al.*, 2012). FtsH proteases as well as FtsHi enzymes have been shown to be very important during plant and chloroplast development (Zaltsman *et al.*, 2005; Liu *et al.*, 2010; Kato *et al.*, 2012; Kato and Sakamoto, 2014; Schreier *et al.*, 2018) and mutants depleted of FtsHi1 or FtsHi4 have been found to be seed-lethal (Kadirjan-Kalbach, *et al.*, 2012; Lu *et al.*, 2014). In our study most *FTSHi* mutants remained heterozygous with gene expressions of 40–60%, whilst *ftshi1-1*, *ftshi3-2*, and *ftshi4-2* were homozygotes but still showed expression at 10–30% of the wild-type (WT) level. These weak homozygous or heterozygote mutants that are not seed-lethal offer an excellent opportunity to investigate the impact of FtsHi enzymes on the plant cell.

Plastid biogenesis has major effects on leaf development as the chloroplast not only serves as the photosynthetic apparatus but also houses several essential metabolic pathways that are important for different aspects of plant growth and development. Plants carrying a mutation in a nuclear-encoded chloroplast protein often show a pale-seedling phenotype (Myouga *et al.*, 2010); indeed, the phenotype of pale-green seedlings and/or variegated leaves is well established for mutants of the thylakoid-located FtsH. In this study we demonstrated that *ftshi1-1* (Kadirjan-Kalbach *et al.*, 2012), *ftshi3-2*, and *ftshi4/FTSHi4-1* displayed a pale-seedling phenotype during the first 14 d after germination (Fig. 4), indicating slow development of the photosynthetic apparatus and/or the thylakoid membrane. Older plants, however, resembled their corresponding WT, both when exposed to stress or grown in the field. Interestingly, a significantly lower total chlorophyll content compared to the WT was observed in seedlings of *ftshi2/FTSHi2* and *ftshi5/FTSHi5* even though they did not display any obvious pale phenotype (Fig. 4, Supplementary Table S7). This finding differs to the data of Wang *et al.* (2018) who studied *RNAi-FtsHi5* plants. Whilst the pale-seedling phenotype of *ftshi3-2* and *ftshi4/FTSHi4-1* was restricted to younger plants, *ftshi1-1* displayed pale leaves throughout its life span, accompanied with a significantly smaller rosette size when grown in the field or exposed to cold or high temperature stress. Notably, when grown under continuous light the leaves of *ftshi1-1* were as green as those of its corresponding WT, and a smaller rosette size was not observed in plants at 6 weeks old. Comparisons of the proteome of *ftshi1-1* and its WT during various growth conditions are in progress.

Besides its pale-seedling phenotype, the growth of *ftshi3-2* was not affected by any of the stress conditions. Interestingly, however, the NPQ of *ftshi3-2* was the most strongly affected of all the *FTSHi* mutants directly after stress exposure. This effect, however, might not only have been caused directly by diminished levels of FtsHi3. AT2G15820, encoding OTP51, a splicing factor essential for the splicing of Ycf3 intron 2, was co-expressed with *FtsHi*3. Loss of OTP51 impacts the assembly of PSI and PSII subunits as well as the photosynthetic fluorescence characteristics of mutant plants (De Longevialle *et al.*, 2008). In our *ftshi3-2* mutant the expression of *OTP51* was up-regulated to compensate for the loss (Fig. 3). While the heterozygote mutant *ftshi4/FTSHi4-1* displayed a pale-seedling phenotype with significantly lower levels of total chlorophyll content (Fig. 4, Supplementary Table S7), which has also been observed in a *ftshi4* mutant constructed by RNAi (Lu *et al.*, 2014), our homozygous *ftshi4-2* mutant displayed a mild phenotype of smaller rosette size then the corresponding WT at the same age, together with lower photosynthetic efficiency (Supplementary Table S6).

Growth under outdoor conditions is the ultimate challenge for plants. Mutants that do not display any phenotype under controlled growth conditions usually have reduced growth and/or Darwinian fitness when exposed to the continually varying conditions of a field trial (Wagner *et al.*, 2011). Comparing the phenotype and rosette size of all *FTSHi* mutants under outdoor field conditions, only *ftshi1-1* displayed an obvious phenotype, which was similar to the one observed in controlled growth (Fig. 4). However, when evaluating the Darwinian fitness of the *FTSHi* mutants, the total number of siliques was significantly reduced not only in *ftshi1-1* but also in *ftshi4*/*FTSHi4-1*, *ftshi4-2*, and *ftshi5/FTSHi5*-1 (Fig. 5). In addition, a significantly lower number of seeds per silique was observed for *ftshi1-1*, *ftshi3-2*, *ftshi4*/*FTSHi4-1*, and *ftshi5/FTSHi5-1*. While we did not detect any significant differences in seed viability between any mutants and their corresponding WTs, a significant delay in seed germination was observed for *ftshi1-1*, *ftshi2*/*FTSHi2*-*6*, *ftshi3-2*, *ftshi4*/*FTSHi4-1*, and *ftshi4-2* (Fig. 6), further lowering the Darwinian fitness of these mutants. The molecular mechanism for these obvious effects remains to be elucidated. The reduced fitness of the *ftshi2*/*ftshi2-6*, *ftshi4*/*FTSHi4-1*, and *ftshi4-2* mutants indicates the importance of these presumably proteolytically inactive FtsH enzymes; as mentioned above, homozygous and strong *ftshi2* and *ftshi4* knock-out mutants are known to be seed-lethal. *Sco* (snowy cotyledon) mutants, the *ex1 ex2* double-mutant (executer1/executer2) and the *spd1* mutant (seedling plastid development 1) are other mutants impaired in chloroplast biogenesis that display obvious phenotypes only at the seedling stage (Lee *et al.*, 2007; Albrecht *et al.*, 2008; Ruppel *et al.*, 2011). Young cotyledons of *sco* contain both normal and aberrant chloroplasts, suggesting that there is a ‘threshold’ of impaired chloroplast development above which normal development still is possible (Albrecht *et al.*, 2010; Tanz *et al.*, 2012). Comparisons of the strong and weak *FTSHi* mutants might provide unique possibilities to understand the regulatory processes responsible for such a ‘gradient’ of normal and impaired chloroplast development during chloroplast biogenesis.

The observed linkage of chloroplast development and plant development (Kadirjan-Kalbach, *et al.*, 2012; Lu *et al.*, 2014; Wang *et al.*, 2018) led us perform *in silico* co-expression analyses with *FTSHi* to find other genes encoding proteins involved either in common pathways or in the regulatory processes of chloroplast biogenesis. Gene expression analysis supported our *in silico* data. *FTSHi2*, *FTSHi5*, and *FTSHi4* were shown to be strongly correlated, while *FTSHi1* was connected to this cluster via its co-expression with *FTSHi4* (Fig. 2). Expression of *FTSHi*5 was additionally found to be strongly correlated with *FTSH12*, which encodes a presumably proteolytically active protease. Transcript levels of *FTSH12* were significantly higher in *ftshi5/FTSHi5-1* and conversely expression of *FTSHi*5 was found to be significantly increased in the *ftsh12* knock-down line (Fig. 3), confirming our prediction from ATTED-II (Supplementary Table 4). These results are in accordance with a recent study by Schreier *et al.* (2018), who were able to co-isolate a FtsH12/FtsHi1,2,4,5 complex that performs co-immunoprecipitation with NAD-dependent malate dehydrogenase (MDH), and also with a study by Kikuchi *et al.* (2018), who isolated a 2-MDa complex containing Ycf2 and FtsH12/FtsHi1,2,4,5. In a hexameric FtsH12/FtsHi complex FtsH12 would be assumed to provide the protease unit while the FtsHi added an effective ATPase. Interestingly, however, it has been shown that the zinc-binding domain in FtsH12 is not crucial for the essential function of protein import into the chloroplast (Kikuchi *et al.*, 2018). It remains to be established whether the different FtsHi subunits in this complex can substitute for each other or if they are assembled during specific environmental conditions. While Kikuchi *et al.* (2018) performed immunoblots with antibodies directed against the different subunits of the complex, differences in concentration and binding-specificity of the antibodies did not allow stoichiometric analyses. Also, FtsHi4 has been detected both in the envelope (Ferro *et al.*, 2010) as well as in the thylakoid membrane (Lu *et al.*, 2014) of the chloroplast. The obvious question remains as to why are so many different (presumably proteolytically inactive) FtsH subunits needed in the envelope membrane? Interestingly, FtsHi3 was not detected in the FtsH12/FtsHi complex, and in our co-expression analyses its corresponding gene had a different expression pattern compared to the other enzymes (Supplementary Table 4). In a phylogenetic analysis using UPGMA (unweighted pair group method with arithmetic mean), FtsHi3 was found to be quite distant to FtsH12 and the other FtsHis (Garcia-Lorenzo *et al.*, 2008). Biochemical studies identified FtsHi3 in a 1-MDa complex distinct from the 2-MDa FtsH12/FtsHi/Ycf2 complex and the 1-MDa TIC (translocon on the inner chloroplast membrane) complex (Kikuchi *et al.*, 2013, 2018). The FtsHi3 enzyme was therefore suggested to form a different import motor complex, which functions redundantly to the main FtsH12/FtsHi complex (Kikuchi *et al.*, 2018).

FtsH proteases emerged early in evolution and are present in organisms ranging from eubacteria to vertebrates. Recently Kikuchi *et al.* (2018) proposed that the chloroplast-encoded Ycf2 contains evolutionary similarity with the presumably proteolytically inactive FtsH-like members. Ycf2 has a putative AAA ATP-binding domain (Wolfe, 1994) and is essential for cell survival (Drescher *et al.*, 2000). Ycf2 belongs to the FtsH12/FtsHi1,2,4,5-Ycf2-NAD-MDH complex, the ATP-driven motor associated with the TIC complex Tic20/Tic56/Tic100/Tic214(Ycf1) that imports pre-proteins across the chloroplast envelopes (Kikuchi *et al.*, 2013, 2018; Nakai, 2015, 2018; Schreier *et al.*, 2018). On the other hand, bacteria belonging to the Firmicutes were found to have paralogs to the FtsH/FtsHi enzymes that were acquired via horizontal gene transfer (Shao *et al.*, 2018). More detailed work is required to determine whether the FtsH12/FtsHi complex and Ycf2 originate from a chloroplast-encoded FtsH of an ancestral endosymbiont (Kikuchi *et al.*, 2018) or from other bacteria via horizontal gene transfer (Shao *et al.*, 2018). FtsHs are important for photosystem repair in cyanobacteria and chloroplasts and multiplied very early, probably accompanying the evolution of photosynthesis (Bailey *et al.*, 2002; Silva *et al.*, 2003; Zaltsman *et al.*, 2005; Komenda *et al.*, 2006; Shao *et al.*, 2018). *Prochlorococcus marinus* SS120, a specialist adapted to low light , contains a minimal oxophototrophic genome but still encodes several *FTSH* gene copies (Fig. 1; Dufresne *et al.*, 2003). In eukaryotic cells FtsH proteins are encoded in the nucleus and after translation are transported to either plastids or mitochondria. A recent study reported five FtsH copies in *Chlamydomonas reinhardtii*, one of which clustered together with Arabidopsis FtsH7/9, an envelope-localized complex in higher plants (Shao *et al.*, 2018). Previously, Malnoë *et al.* (2014) detected nine nuclear-encoded FtsHs, including six active and three presumably inactive versions (FtsH1, FtsH2, FtsH3, FtsH4, FtsH7, FtsH11, FHL1, FHL2, FHL3). Therefore, green algae might have already utilized multiple FtsHs for maintenance and/or import over their inner envelope. However, the FtsH gene copy number increases drastically with multicellular land plants with true tissue and organs (Fig. 1). FtsHs with increased similarities to FtsH12 had already emerged in mosses. In eudicots this envelope-located FtsH12-FtsHi complex seems to be necessary for viability. FtsH12-FtsHi, located in the plastid envelope, is known to be involved in plastid differentiation (Kadirjan-Kalbach *et al.*, 2012; Lu *et al.*, 2014; Wang *et al.*, 2018) and import (Schreier *et al.*, 2018; Kikuchi *et al.*, 2018). This phylogenetic group also shows the highest copy numbers of active as well as presumably proteolytically inactive FtsH enzymes. Two phylogenetic groups have replaced such an FtsH12-FtsHi complex: gymnosperms and grasses. Apart from keeping a high copy number of FtsHs for maintenance of the photosystems and the respiratory chain, they contain neither genes with high sequence similarity to FtsH12 nor multiple copies of FtsHi. In monocotyledons, Ycf2 additionally seems to be absent (Fig. 1), indicating that they use different TIC import motors (see also Nakai, 2018).

*FTSHi2*, *FTSHi4*, and *FTSH12* also showed strong co-expression with *TAC12*/*HEMERA* (Fig. 2), and its corresponding protein is localized both in the plastidic transcriptional apparatus as well as in the nucleus (Pogson *et al.*, 2008; Chen *et al.*, 2010). The dual localization of TAC12 makes it an interesting candidate for retrograde signaling during chloroplast development. TAC12 could regulate the nuclear gene transcription during plastid biogenesis and therefore be affected in mutants of *FTSHi2*, *FTSHi4*, and *FTSH12*. *FTSHi2* additionally was strongly co-expressed with *mraW* methylase family protein, which clusters with *FTSHi3* via *OTP51*. MraW has been found to be located in chloroplasts (Dal Bosco *et al.*, 2004), although its function is unknown. Transcript levels of *mraW* were significantly higher in *ftshi2*/*ftshi2-5* than in the WT, and the expression of *OTP51* was higher in *ftshi2*/*ftshi2-5* compared to the WT (Fig. 3). However, the expression of *OTP51* and *mraW* did not change significantly in *ftshi3-2*. The strongest impact on the expression of all the genes investigated was visible in 6-week-old plants of the weak homozygous mutant *ftshi1-1*, with expression of *FTSHi*s, *FTSH12*, *OTP51*, and *mraW* being found to be significantly lower than in the WT. Down-regulation of these genes could be a result of the pleiotropic effect of under-developed chloroplasts in these mutants; alternatively, these genes could be regulated downstream of *FTSHi1*. Conversely, the expression of *FTSHi1* was down-regulated in all *FTSHi* mutants, with significantly lower expression of *FTSHi1* and *FtsH12* in comparison to the WT being observed in *ftshi3-2* and *ftshi4-2*. The FtsHi3 and FtsHi4 subunits therefore seem not to be able to substitute for the lower amount of FtsHi1. In the *ftshi12* knock-down mutant, expression of all *FTSHi* genes was up-regulated to ~120% of the WT value.

In conclusion, we found that FtsHi enzymes have evolved concomitantly with FtsH12 in flowering plants, specifically in eudicots. While an envelope-located, hexameric complex of FtsH12/FtsHi1,2,4,5 is supported by our expression and co-expression data, FtsHi3 seems to be involved in other functions and might form a complex with FtsH7 and/or FtsH 9. Reduced expression of *FTSHi1*, *3*, and *4* leads to a pale-seedling phenotype. While no obvious phenotype was visible in older plants, even during exposure to stress, the Darwinian fitness was affected in these plants.

## Supplementary data

Supplementary data are available at *JXB* online.

Fig. S1. Predicted domains and motifs in AtFtsHis in comparison to the presumably active AtFtsH12.

Fig. S2. Schematic diagram of the five *FTSHi* genes.

Fig. S3. Phenotypes of homozygous *ftshi1-1*, *ftshi3-2*, and *ftshi4-2* mutants exposed to stress conditions.

Fig. S4. Summary of the light and temperature conditions in the outdoor experiment.

Table S1. List of *FTSHi* mutant lines and their corresponding NASC numbers, and the T-DNA insertion type and its location.

Table S2. Primers used for genotyping and q-PCR.

Table S3. Results from blastp with full-length FtsHi1.

Table S4. Genes co-expressed with *FTSHi*, and their corresponding proteins and localizations predicted by TargetP and WoLF PSORT as identified in ATTED-II.

Table S5. Genotypes of the *FTSHi* mutants, the reduction of their gene expression compared to the wild-type, and the phenotype of seedlings at 8 d old.

Table S6. Chlorophyll fluorescence parameters for 2-week-old homozygous *FTSHi* mutants exposed to stress conditions.

Table S7. Chlorophyll contents and chlorophyll *a*/*b* ratios of 2-week-old seedlings.

Table S8. Chlorophyll fluorescence parameters for 45-d-old field-grown plants.

Supplementary Figures and Tables

## Abbreviations

FtsH: filamentation-temperature-sensitive protein H
mraW: (S-adenosyl-L-methionine-dependent methyltransferase) methylase family protein
OTP51: organelle transcript processing 51
PAM: pulse-amplitude modulation
arc1: accumulation and replication of chloroplasts 1
*F*_v_/*F*_m_: maximum potential quantum efficiency of PSII
NPQ: non-photochemical quenching.
