# Supplementary material for: Reduced expression of the proteolytically inactive FtsH members has impacts on the Darwinian fitness of Arabidopsis thaliana
Source: J Exp Bot. 2019 Feb 4;70(7):2173–84. doi: 10.1093/jxb/erz004 (PMC6460958; doi:10.1093/jxb/erz004)
Supplement: Supplementary Figures and Tables [file erz004_supplementary-figures-s1-s4_tables-s1-s8.pdf]

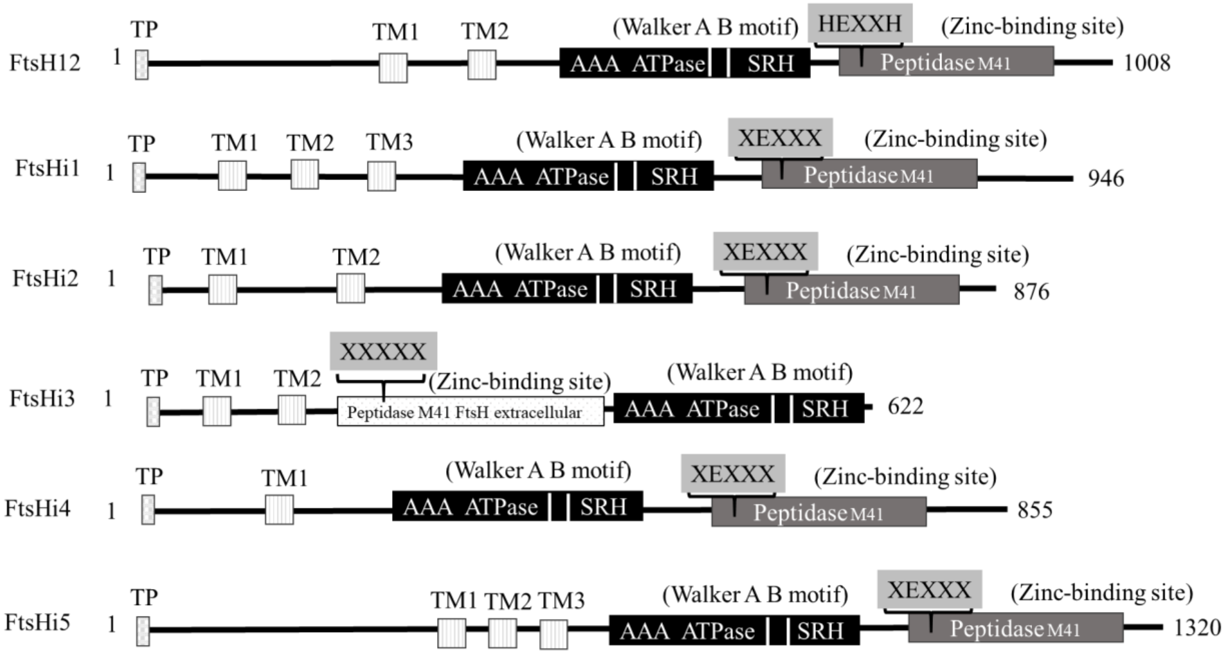

Supplementary Figure 1. Predicted domains and motifs in AtFtsHi´s in comparison to the presumably active AtFtsH12. TP, transit peptide; transmembrane domains, TM1-3; Walker A B motifs are indicated as two white lines between the AAA ATPase and SRH; SRH, Second region of homology. Active FtsH proteases contain the Zn^{2+}-binding motif (HEXXH) in their peptidase M41 domain, which is substituted or absent in presumably inactive FtsHi's (substitution of both histidines indicated as XEXXX). In FtsHi3 the peptidase M41 domain is annotated as "FtsH is extracellular" and located N-terminal to the ATPase, its HEXXH motif is completely missing.

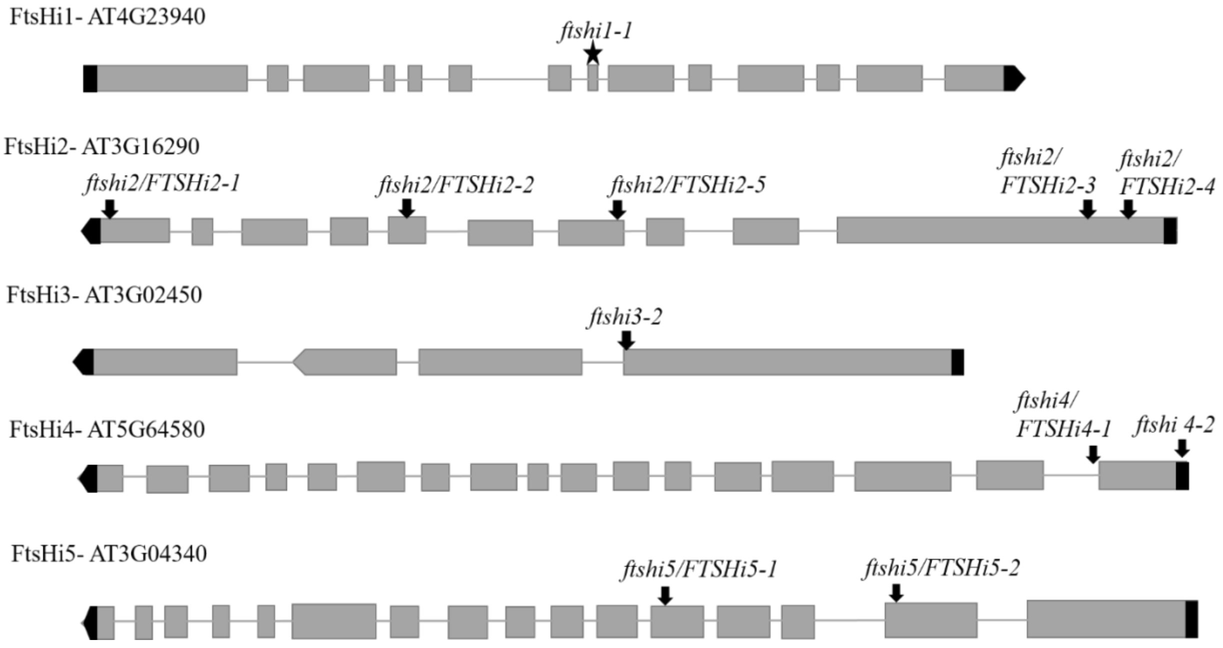

Supplementary Figure 2. Schematic diagram of the five *FTSHi* genes, indicating the site of mutation (*ftshi1-1*) or the location of the T-DNA insertion. Highlighted in black are the UTR's.

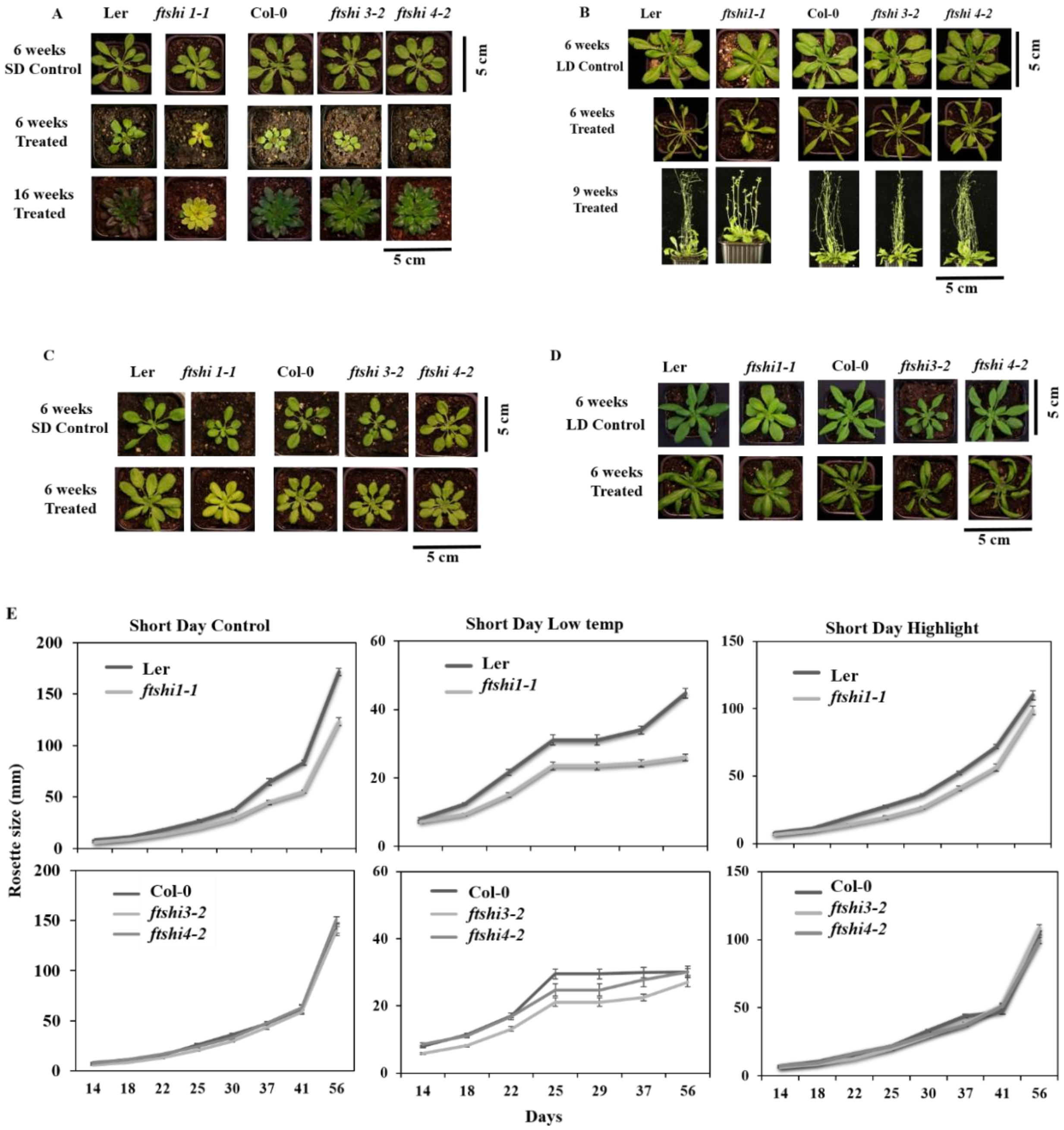
A
Ler
ftshi 1-1
Col-0
ftshi 3-2
ftshi 4-2
6 weeks SD Control
6 weeks Treated
16 weeks Treated
5 cm
5 cm
B
Ler
ftshi1-1
Col-0
ftshi 3-2
ftshi 4-2
6 weeks LD Control
6 weeks Treated
9 weeks Treated
5 cm
5 cm
C
Ler
ftshi 1-1
Col-0
ftshi 3-2
ftshi 4-2
6 weeks SD Control
6 weeks Treated
5 cm
5 cm
D
Ler
ftshi1-1
Col-0
ftshi3-2
ftshi 4-2
6 weeks LD Control
6 weeks Treated
5 cm
5 cm
E
Short Day Control
Short Day Low temp
Short Day Highlight
Ler
ftshi1-1
Col-0
ftshi3-2
ftshi4-2
Rosette size (mm)
Days
14 18 22 25 30 37 41 56
14 18 22 25 29 37 56

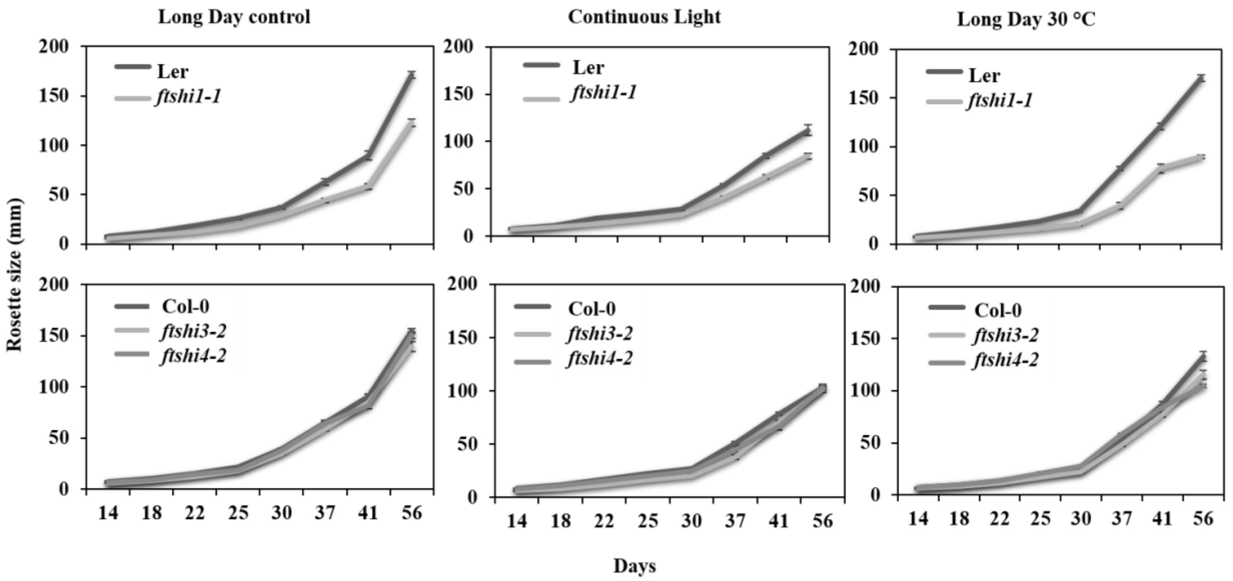

Supplementary Figure 3. Phenotypes of homozygous *ftshi1-1, ftshi3-2,* and *ftshi4-2* mutants, which had been exposed to stress conditions from the age of 2 weeks. **(A)** Exposure to short day (SD) and cold temperature (4 °C); (**B)** long day (LD) and high temperature (30 °C); **(C)** SD and highlight (700 µmol photons m^{-2} s^{-1}); **(D)** continuous light (150 µmol photons m^{-2} s^{-1}). **(E)** Rosette diameter measurements were performed from week 2 to 6 on short day control (right panel), short day low temperature (middle panel) and short day highlight (left panel), long day control (left panel), continuous light (middle panel) and long day 30 °C (right panel).

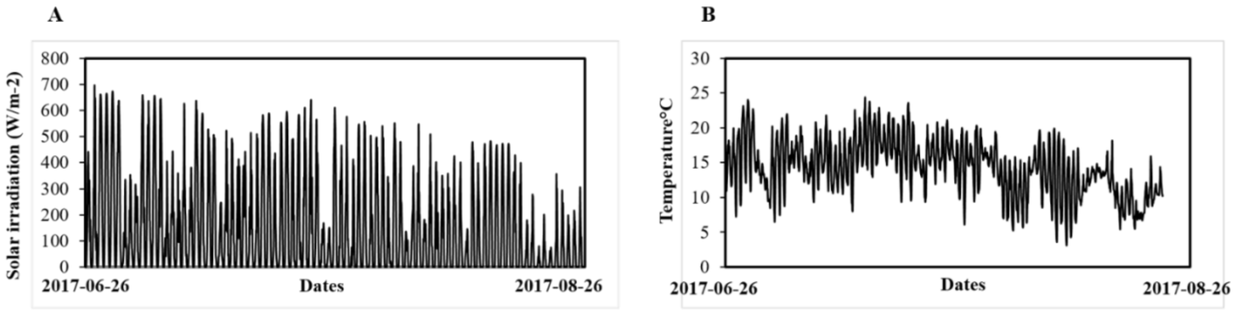

Supplementary Figure 4: Light (A) and temperature (B) conditions in the field 63°49'07.2"N 20°18'45.0"E in summer 2017. Weather data are available at http://www8.tfe.umu.se/weather-new/hamta_vaderdata.html.

Supplementary Table 1: Collection of the *FTSHi* mutant lines and their corresponding NASC ID's, t-DNA insertion type and location

NASC ID	Antibiotic selection		Gene name	t-DNA insertion	Location
N1298		Ler			
CS16208		Col-0			
N907		Col-2			
N908		Col-3			
N262	EMS	*ftshi 1-1*	AT4G23940	Missense mutation	Exon
N679694	KAN	*ftshi2/FTSHi2-1*	AT3G16290	SALK_054712C	Exon
CS853725	BAR	*ftshi2/FTSHi2-2*	AT3G16290	WISCDSLOX383F4	Exon
CS16208	BAR	*ftshi2/FTSHi2-3*	AT3G16290	emb2083-3	Exon
CS16209	BAR	*ftshi2/FTSHi2-4*	AT3G16290	emb2083-4	Exon
CS16167	BAR	*ftshi2/FTSHi2-5*	AT3G16290	emb2083-2	Exon
N843617	BAR	*ftshi2/FTSHi2-6*	AT3G16291	SAIL_1178_A11	1000-Promotor
N469342	SUL	*ftshi3-2*	AT3G02450	GK-723C06_025364	Exon
N436594	SUL	*ftshi4/FTSHi4-1*	AT5G64580	GK-382B06	Intron
N675639	KAN	*ftshi4-2*	AT5G64580	SALK_067969.20.10.x	300-UTR5
N613657	KAN	*ftshi4/FTSHi4-3*	AT5G64580	SALK_113657	Intron
CS16181	BAR	*ftshi5/FTSHi5-1*	AT3G04340	GK-058A04	Exon
N405476	SUL	*ftshi5/FTSHi5-2*	AT3G04340	emb2458	Exon

Supplementary Table 2: Primers used for genotyping and q-PCR.

Number	Mutant	Primer name	Primer sequence	Purpose
1		LbB1.3	ATTTTGCCGATTTCGGAAC	T-DNA primer
2		P745	AACGTCCGCAATGTGTTATTAAGTTGTC	T-DNA primer
3		LB3	TAGCATCTGAATTTCATAACCAATCTCGATACAC	T-DNA primer
4		o8409	ATATTGACCATCATACTCATTGC	T-DNA primer
5		o8474	ATAATAACGCTGCGGACATCTACATTTT	T-DNA primer
6		Arc1-seqF	AGTTGGGTCAGCTCGTATCAG	Sequencing
7		arc-1F	TGGAGTTGGGTCAGCTCGTA	Forward primer, PCR
8	*ftshi1-1*	arc-1R	TGGCTTGCGATTGTCGTTTG	Reverse primer, PCR
9		i2_1F	GATATTGATGTGGAGGCATTG	Forward primer, PCR
10	*ftshi 2-1*	i2_1R	ATTAAGCCATTAAGGTGGCCA	Reverse primer, PCR
11		i2-2F	AAGCTGCAATGGCTGTTGT	Forward primer, PCR
12	*ftshi 2-2*	i2-2R	AAATTAAAATAATCCACATGCTCCA	Reverse primer, PCR
13		i2-3F	ATGCAGAGGGAAACGACAAGCCAT	Forward primer, PCR
14	*ftshi 2-3*	i2-3R	TGGAATCTTGACCCCTCTC	Reverse primer, PCR
15		i2-4F	CTACTTCAATTGAATCACATCATG	Forward primer, PCR
16	*ftshi 2-4*	i2-4R	GCCATATCTCGATAATTTTTCCT	Reverse primer, PCR
17		i2-5F	TCTTCTCTATCTCTGCTTCTC	Forward primer, PCR
18	*ftshi 2-5*	i2-5R	CTGCTCCAACCATGCCATCTG	Reverse primer, PCR
19		i2_SAIL_F	AGAGATGTACCGCAGGAGAGG	Forward primer, PCR
20	*ftshi 2-6*	i2_SAIL_R	CCACCTCTGTTCTTCCATCAC	Reverse primer, PCR
21	*ftshi 3-2*	*i3-2F*	TAGTTGCAGGAGACTTGGTGG	Forward primer, PCR
22		i3-2R	GGAAGGTGAGTTTTTCCTTGC	Reverse primer, PCR
23	*ftshi 4-1*	i4_GK_F	CATTTCTAAACATACCGACTCTTG	Forward primer, PCR
24		i4_GK_R	CTTAAACCCTTCTAATACCCTCTT	Reverse primer, PCR
25	*ftshi 4-2*	i4-salk067969_2F	CGATCAAAGTCCGAAACTGAG	Forward primer, PCR
26		i4-salk067969_2R	CTCTGCCTAAGATCGCATTTG	Reverse primer, PCR
27	*ftshi 4-3*	i4-salk113657_3F	CATTCCCGTCTGAAGAATCAG	Forward primer, PCR
28		i4-salk113657_3R	GAGCAGAACTGCAAAACGTTC	Reverse primer, PCR
29		i5-1F	GCCACAACCTCGTTGATTTCTTCT	Forward primer, PCR
30	*ftshi5-1*	i5-1R	GCCAGGATTAGGCATGATTATGC	Reverse primer, PCR
31		i5-2F	ATGCTAGAGATCAAGAGTCAACTA	Forward primer, PCR
32	*ftshi5-2*	i5-2R	ATAGCATACATTCGTCCATACTAC	Reverse primer, PCR
33		qi-1-F	ATTCCTCGTGGTCAGACAC	Forward primer, q-PCR
34	*ftshi1*	qi-1-R	CTCCGAGCAAGACCTGAAG	Reverse primer, q-PCR
35		qi-2-F	GCAGGTTCATGCTCGTAAGA	Forward primer, q-PCR
36	*ftshi 2*	qi-2-R	GCTCCAACCATGCCATCTG	Reverse primer, q-PCR
37		qi-3-F	CCAGACGTTAAACCAGTTGC	Forward primer, q-PCR
38	*ftshi3*	qi-3-R	CTTCTGGTCGGTTAGTTGC	Reverse primer, q-PCR
39		qi-4-F	GCATGTGCTCCTAGAGTAGT	Forward primer, q-PCR
40	*ftshi4*	qi-4-R	CCGCTCGCTGTGAAGCT	Reverse primer, q-PCR
41		qi-5-F	GATTGGCGTAAGGTTTCAGAG	Forward primer, q-PCR
42	*ftshi5*	qi-5-R	ATGGCTAAACGTCGCAAACC	Reverse primer, q-PCR
43		ACT2-F	CTTGCACCAAGCAGCATGAA	Forward primer, q-PCR
44	ACT	ACT2-R	CCGATCCAGACACTGTACTTCCTT	Reverse primer, q-PCR
45		UBQ5-F	ACGCTTCATCTCGTC	Forward primer, q-PCR
46	UBQ5	UBQ5-R	CCACAGGTTGCGTTA	Reverse primer, q-PCR

Supplementary Table 3: Blastp with Full Length FtsHi1 (O22993)

	Top Score	bits	e	cover	identity (%)	top hit inactive?	No. of inactive
***A. thaliana* (TAIR10)**	1612.8	4178	0	945	100	Y	5
Chlamydomonas rheinhardtii	259.6	662	5.20E-74	532	38	N	3*
Dunaliella salina	256.1	653	6.30E-73	526	36.9	N	2
Physcomitrella patens	742.7	1916	0	878	51.5	Y	4
Selaginella moellendorffii	659.4	1700	0	881	48.1	Y	3
Spirodela polyrhiza	1089.3	2816	0	860	72.4	Y	6
Oryza sativa	263.1	671	3.90E-75	495	39	N	1**
Setaria italica	259.2	661	7.10E-74	493	38.5	N	1
Zea mays PH207	255	650	3.10E-72	493	37.1	N	1
Aquilegia coerulea	1080.5	2793	0	892	70	Y	7
Populus trichocarpa	1191.4	3081	0	944	74.8	Y	4
Solanum lycopersicum	1154.8	2986	0	857	77.5	Y	5
Glycine max	1154.8	2986	0	924	73.5	Y	16
Synechocystis sp. PCC6803	337	864	1.00E-104	533	39	N	0
Prochlorococcus marinus SS120	340	872	2.00E-104	526	39	N	1
Anabaena cylindrica	340	873	3.00E-104	545	38	N	0
Picea abies	417.5	1072	1.20E-135	391	51	Y*	1
						*Does not cover Protease domain	*Malnoë et al., 2014
							**Garcia et al., 2008

Supplementary Table 4. Genes co-expressed with *FTSHi*, and their corresponding proteins and localizations predicted by TargetP and WoLF PSORT as identified in ATTED-II

Protein name	Gene name	target_TargetP	target_WoLF	Reference
FTSHI5	At3g04340	Chloroplast	chloroplast	Ferro *et al.,* 2010
FTSHI3	At3g02450	Chloroplast	plasma membrane	Ferro *et al.,* 2010
TAC12	At2g34640	Chloroplast	nuclear	Chen *et al.,* 2010
FTSHI1	At4g23940	Chloroplast	chloroplast	Kadirjan-Kalbach *et al.*, 2012
FTSHI4	At5g64580	Chloroplast	chloroplast	Lu *et al.,* 2014
EMB1270	At3g18110	Chloroplast	chloroplast	Cushing *et al.,* 2005
OVA2	At5g49030	Chloroplast	chloroplast	Berg *et al.,* 2005
FTSHI2	At3g16290	Chloroplast	chloroplast	Lu *et al.,* 2014
OTP51	At2g15820	Chloroplast	chloroplast	De Longevialle *et al.*, 2008
SRF1	At2g20850	Secretory pathway	vacuole	Tanaka *et al.,* 2009
GLR5	At2g32400	Secretory pathway	plasma membrane	Michard *et al.,* 2011
METHYLASE	At5g10910	Chloroplast	chloroplast	Dal Bosco *et al.,* 2004
FTSH12	At1g79560	Chloroplast	chloroplast	Schreier *et al.,* 2018

Additional References

Cushing DA, Forsthoefel NR, Gestaut DR, Vernon DM. 2005. Arabidopsis emb175 and other ppr knockout mutants reveal essential roles for pentatricopeptide repeat (PPR) proteins in plant embryogenesis. *Planta* **221**, 424-436.

Michard E, Lima PT, Borges F, Silva AC, Portes MT, Carvalho JE, Gilliham M, Liu L-H, Obermeyer G, Feijó JA. 2011. Glutamate receptor–like genes form Ca2+ channels in pollen tubes and are regulated by pistil D-serine. *Science*, **332**, 434-437.

Pastuglia M, Azimzadeh J, Goussot M, Camilleri C, Belcram K, Evrard J-L, Schmit A-C, Guerche P, Bouchez D. 2006. γ-Tubulin is essential for microtubule organization and development in Arabidopsis. The Plant Cell **18**, 1412-1425.

Tanaka M, Takahata Y, Nakayama H, Nakatani M, Tahara M. 2009. Altered carbohydrate metabolism in the storage roots of sweetpotato plants overexpressing the SRF1 gene, which encodes a Dof zinc finger transcription factor. *Planta* **230**, 737-746.

Supplementary Table 5: Genotypes of the *FTSHi* mutants, reduction of their gene expression compared to WT (given in %) and their phenotype at 8 days seedling stage.

Line	Genotype	Gene expression reduced by %	Phenotype
			Seedlings on plate
ftshi 1-1	HM	88 ± 1.1	Pale
ftshi2/FTSHi2-5	HT	62 ± 1.5	No change
ftshi2/FTSHi2-6	HT	35 ± 0.8	No change
ftshi 3-2	HM	90 ± 0.2	Pale
ftshi4/FTSHi4-1	HT	53 ± 1.1	Pale
ftshi 4-2	HM	61 ± 4.8	No change
ftshi5/FTSHi5-1	HT	63 ± 2.7	No change
ftshi5/FTSHi5-2	HT	36 ± 0.2	No change

Supplementary Table 6. Chlorophyll fluorescence parameters for 2-week-old homozygous *FTSHi* mutants exposed to stress conditions. Measurements were performed at room temperature during steady-state conditions on homozygote *FTSHi* mutants exposed to stress conditions. Measurements were performed at t0 and 3 d, 6 weeks (and 16 weeks in cold stress). High light (HL) 700 μmol photons m^{-2} s^{-1}; continuous light (CL): 150 μmol photons m^{-2} s^{-1}. Asterisks indicate P<0.05 according to ANOVA and a least-significant difference (LSD) *post hoc* test.

**SD/4°C**	**0 Hr**		**3 Day**		**6 Weeks**		**16 Weeks**	
**Genotype**	**FV/FM**	**NPQ**	**FV/FM**	**NPQ**	**FV/FM**	**NPQ**	**FV/FM**	**NPQ**
**Ler**	**0.8 ± 0.002**	**1.6 ± 0.12**	**0.61 ± 0.01**	**0.82 ± 0.07**	**0.73 ± 0.00**	**0.97± 0.07**	**0.74 ± 0.00**	**1.23 ± 0.09**
ftshi 1-1	**0.8 ± 0.003**	**1.3* ± 0.07**	**0.63 ± 0.00**	**0.84 ± 0.10**	**0.64 ± 0.01**	**1.07 ± 0.07**	**0.71 ± 0.01**	**1.15 ± 0.04**
**Col-0**	**0.8 ± 0.003**	**1.1 ± 0.06**	**0.61 ± 0.01**	**0.88 ± 0.04**	**0.73 ± 0.00**	**1.01 ± 0.11**	**0.78 ± 0.00**	**0.79 ± 0.05**
ftshi 3-2	**0.8 ± 0.003**	**1.0 ± 0.10**	**0.60 ± 0.02**	**0.71*± 0.05**	**0.73 ± 0.00**	**1.21 ± 0.10**	**0.71 ± 0.00**	**1.08 * ± 0.08**
ftshi 4-2	**0.8 ± 0.005**	**1.2 ± 0.07**	**0.58 ± 0.01**	**0.78 ± 0.07**	**0.75 ± 0.00**	**1.38* ± 0.05**	**0.75 ± 0.00**	**0.6 * ± 0.08**
**LD/30 °C**	**0 Hr**		**3 Day**		**6 Weeks**		**16 Weeks**	
**Genotype**	**FV/FM**	**NPQ**	**FV/FM**	**NPQ**	**FV/FM**	**NPQ**	**FV/FM**	**NPQ**
**Ler**	**0.8 ± 0.002**	**1.6 ± 0.12**	**0.70 ± 0.01**	**1.15 ± 0.05**	**0.70 ± 0.01**	**1.18± 0.05**	**n.d.**	
ftshi 1-1	**0.8 ± 0.003**	**1.3 ± 0.07**	**0.63 ± 0.01**	**1.01 ± 0.06**	**0.70 ± 0.01**	**1.23 ± 0.06**	**n.d.**	
**Col-0**	**0.8 ± 0.003**	**1.1 ± 0.06**	**0.71 ± 0.00**	**1.29 ± 0.09**	**0.70 ± 0.01**	**1.19 ± 0.05**	**n.d.**	
ftshi 3-2	**0.8 ± 0.003**	**1.0 ± 0.10**	**0.68 ± 0.00**	**0.98* ± 0.04**	**0.67 ± 0.01**	**1.45 ± 0.11**	**n.d.**	
ftshi 4-2	**0.8 ± 0.005**	**1.2 ± 0.07**	**0.69 ± 0.00**	**1.02 ± 0.08**	**0.71 ± 0.00**	**0.98 * ± 0.08**	**n.d.**	
**CL**	**0 Hr**		**3 Day**		**6 Weeks**		**16 Weeks**	
**Genotype**	**FV/FM**	**NPQ**	**FV/FM**	**NPQ**	**FV/FM**	**NPQ**	**FV/FM**	**NPQ**

Ler	**0.8 ± 0.002**	**1.6 ± 0.12**	**0.68 ± 0.02**	**1.03 ± 0.07**	**0.69 ± 0.01**	**1.16± 0.06**	**n.d.**	
ftshi 1-1	**0.8 ± 0.003**	**1.3 ± 0.07**	**0.66 ± 0.02**	**1.06 ± 0.07**	**0.64 ± 0.01**	**0.54 ± 0.12**	**n.d.**	
**Col-0**	**0.8 ± 0.003**	**1.1 ± 0.06**	**0.66 ± 0.01**	**1.03 ± 0.04**	**0.77 ± 0.01**	**0.60 ± 0.05**	**n.d.**	
ftshi 3-2	**0.8 ± 0.003**	**1.0 ± 0.10**	**0.69 ± 0.00**	**0.78* ± 0.04**	**0.73 ± 0.01**	**0.89 ± 0.06**	**n.d.**	
ftshi 4-2	**0.8 ± 0.005**	**1.2 ± 0.07**	**0.68 ± 0.00**	**1.04 ± 0.05**	**0.75 ± 0.00**	**0.98 *± 0.08**	**n.d.**	
**SD/HL**	**0 Hr**		**3 Day**		**6 Weeks**		**16 Weeks**	
**Genotype**	**FV/FM**	**NPQ**	**FV/FM**	**NPQ**	**FV/FM**	**NPQ**	**FV/FM**	**NPQ**
**Ler**	**0.8 ± 0.002**	**1.6 ± 0.12**	**0.70 ± 0.00**	**1.24 ± 0.09**	**0.73 ± 0.01**	**0.91± 0.05**	**n.d.**	
ftshi 1-1	**0.8 ± 0.003**	**1.3 ± 0.07**	**0.62 ± 0.01**	**1.00 ± 0.05**	**0.73 ± 0.01**	**0.87 ± 0.03**	**n.d.**	
**Col-0**	**0.8 ± 0.003**	**1.1 ± 0.06**	**0.70 ± 0.01**	**1.27 ± 0.11**	**0.74 ± 0.01**	**0.59 ± 0.04**	**n.d.**	
ftshi 3-2	**0.8 ± 0.003**	**1.0 ± 0.10**	**0.65 ± 0.00**	**1.17 ± 0.06**	**0.71 ± 0.01**	**0.64 ± 0.06**	**n.d.**	
ftshi 4-2	**0.8 ± 0.005**	**1.2 ± 0.07**	**0.68 ± 0.00**	**1.28 ± 0.08**	**0.73 ± 0.01**	**0.66 * ± 0.07**	**n.d.**	

Supplementary Table 7: Chlorophyll contents and chlorophyll *a*/*b* ratios of 2 weeks old seedlings. Values are given in µg chlorophyll per mg fresh weight. Asterisk indicates a p value of less than 0.05. ANOVA and a least significant difference (LSD) post hoc test and student's *t*-test were performed to determine the statistical significance.

**Genotype**	**Chl *a*+*b* (µg/mg FW)**	**Chl *a*/*b***
Ler	0.85 ± 0.11	3.04 ± 0.02
ftshi 1-1	0.75 ± 0.05*	3.34 ± 0.06*
Col-3	0.82 ± 0.1	3.49 ± 0.05
ftshi2/FTSHi2-6	0.7 ± 0.05	3.42 ± 0.04
Col-0	0.94 ± 0.03	3.04 ± 0.02
ftshi2/FTSHi2-5	0.71 ± 0.09*	3.46 ± 0.05*
ftshi 3-2	0.64 ± 0.03*	3.03 ± 0.08
ftshi4/FTSHi4-1	0.69 ± 0.16*	3 ± 0.07
ftshi 4-2	0.83 ± 0.06*	3.01 ± 0.06
ftshi5/FTSHi5-1	0.74 ± 0.03*	3.46 ± 0.04*
ftshi5/FTSHi5-2	0.68 ± 0.08*	3.27 ± 0.33

Supplementary Table 8. Chlorophyll fluorescence parameters for 45-d-old field-grown plants. Comparison of chlorophyll fluorescence parameters measured during steady-state conditions of field-grown plants. The asterisk indicates a *P*<0.05 according to ANOVA and a least-significant difference (LSD) *post hoc* test.

Genotype	FV/FM	NPQ
**Ler**	**0.79 ± 0.01**	**1.4 ± 0.1**
ftshi1-1	**0.78 ± 0.00**	**0.9* ± 0.1**
**Col-3**	**0.78 ± 0.00**	**1.3 ± 0.1**
ftshi2/FTSHi2-6	**0.79 ± 0.00**	**1.4 ± 0.1**
**Col-0**	**0.78 ± 0.00**	**1.3 ± 0.0**
ftshi2/FTSHi2-5	**0.79 ± 0.00**	**1.3 ± 0.07**
ftshi 3-2	**0.79 ± 0.02**	**1.2 ± 0.1**
ftshi4/FTSHi4-1	**0.78 ± 0.01**	**1.3 ± 0.08**
ftshi 4-2	**0.79 ± 0.07**	**1.4 ± 0.08**
ftshi5/FTSHi5-1	**0.79 ± 0.01**	**1.2 ± 0.2**
ftshi5/FTSHi5-2	**0.81 ± 0.05**	**1.2 ± 0.12**
